# An Eco-Friendly, Interference, and Solvent Free Surfactant-Assisted Dual-Wavelength *β*-CorrectionSpectrometric Method for Total Determination and Speciation of Cu^2+^ Ions in Water

**DOI:** 10.1155/2023/5001869

**Published:** 2023-11-04

**Authors:** Hassan Alwael, Doaa S. Al-Raimi, Khairia M. Al-Ahmary, Hany A. Nasef, Amal S. Alharthi, Tharawat N. Abduljabbar, Liyakat H. Mujawar, Ekram Y. Danish, Mohammad T. Soomro, Mohammad S. El-Shahawi

**Affiliations:** ^1^Department of Chemistry, Faculty of Science, King Abdulaziz University, P.O. Box 80203, Jeddah 21589, Saudi Arabia; ^2^Department of Chemistry, College of Science, University of Jeddah, Jeddah, Saudi Arabia; ^3^Department of Basic Sciences, ta Higher Institute for Engineering and Technology, Mansoura 35111, Egypt; ^4^Center of Excellence in Environmental Studies, King Abdulaziz University, P.O. Box 80216, Jeddah 21589, Saudi Arabia

## Abstract

Spectral interference through the presence of uninformative variables, excess reagents, and complications in the refinement of the analyte signal is common in the quest to identify complex species in real samples. Therefore, an economical green, facile, and sensitive strategy has been developed for Cu^2+^ detection using the anionic surfactant sodium dodecylsulphate- (SDS-) assisted dual-wavelength *β*-correction spectrophotometric strategy combined with the chromogenic reagent zincon (ZI). The low limits of detection (LOD) and quantification (LOQ) of Cu^2+^ using ordinary (single wavelength) spectrophotometry were 0.19 (3.02) and 0.63 (10.0) *μ*gmL^−1^, and these values were improved to 0.08 (1.27) and 0.26 *μ*gmL^−1^ (4.12 *μ*M)) using *β*-correction (dual wavelength) spectrophotometry, respectively. The LOD and LOQ were improved from 0.08 (1.27) and 0.26 (4.12) *μ*gmL^−1^ to 0.02 (0.32) and 0.08 *μ*gmL^−1^ (1.27 *μ*M) using SDS-assisted dual-*β*-correction spectrometry, respectively. Ringbom, *s*, and the corrected absorbance (*A*_*c*_) versus Cu^2+^ concentration plots were linear over the concentration range 1.10–2.4 (17.4–38.1) and 0.50–2.40 *μ*gmL^−1^ (7.94–38.1 *μ*M), respectively. Sandell's sensitivity index of 3.0 × 10^−3^ *μ*g/cm^2^ was achieved. The selectivity was further confirmed via monitoring the impact of common diverse ions and surfactants on the corrected absorbance. Total determination and Cu^2+^ speciation in water were favorably implemented and validated by ICP-OES at 95% (*P*=0.05). Satisfactory Cu^2+^ recoveries in tap (92.2–98.0%) and mineral (105–111.0%) water samples were achieved. The sensing system is simple, reliable, sensitive, and selective for Cu^2+^ detection.

## 1. Introduction

Copper is a crucial micronutrient for phytoplankton and in the human body, and it is an important component of human proteins and enzymes, where the lack of Cu^2+^ will hinder the physiological activities of the human body and easily cause various diseases [1–3]. Chemical speciation (labile and chelated) of copper (II) also plays a strong role in defining the bioavailability and toxicity upon exposure of copper in marine environments. In addition, Cu is a heavy metal widely discovered in the environment and excessive Cu^2+^ ions in water causes severe environmental pollution and even risk of the health of organisms [4]. Furthermore, Cu^2+^ ions beyond the recommended levels of copper causes adverse health effects, e.g., Alzheimer's disease and numerous neurological sicknesses [5]. Industrial and agricultural anthropogenic activities were responsible for a dramatically impact on the environment and human health. Maximum allowable limit (MAL) of copper contamination has been set at 1.3 mg·kg^−1^ (∼20 *μ*M) by the United States-Environmental Protection Agency (US-EPA) and the World Health Organization (WHO) in drinking water and food staffs' regulations [5, 6]. Thus, searching for low-cost, sensitive, and precise reagent for total determination and speciation of Cu^2+^ in water is of great concern [7, 8].

Nowadays, numerous methods based on zincon reagent, i.e., 2-carboxy-2′-hydroxy-5′sulfo-formazyl benzene monosodium salt (*Electronic Supporting Information's*, ESI.  1) [9, 10], solid phase [11–20], cloud point [21–23], ionic liquid-based aqueous two-phase system [24], and dual-ligand Eu-MOF for fluorescence sensing [25] have been reported. Ligandless reversed-phase switchable-hydrophilicity solvent liquid-liquid microextractions combined with atomic absorption spectrometry [26] have also been reported for the detection of Cu^2+^ and other metal ions. Electrothermal atomic absorption spectrometry [27, 28], inductively coupled plasma optical emission spectrometry (ICP-OES) [29], colorimetric [30–33], atomic fluorescence spectrometry [34–37], lighting up of carbon dots [38], and stripping voltammetry [39, 40] have also been reported for Cu^2+^ detection. Most of these techniques have several limitations such as time-consuming, required special sample preparation, highly sophisticated instruments, and high-skill operators [23–27, 33–40].

Ordinary spectrophotometric methods for the detection of metal ions including Cu^2+^ have many advantages such as simplicity, e, applicability, availability, easy to use, and low cost [41–51]. However, the excess of the chromogenic reagent minimizes the sensitivity and precision and limits the linear range of concentration of these methods because of the substantial interfering of the extra concentration of the colored reagent with the analyte at *λ*_max_ [52, 53]. In contrast, the dual-wave *β*-correction spectrophotometric technique has gained great attention and has a promising impact as an alternative approach due to its simplicity, low cost, portability, and elimination of the interference of the excess colorant chromogenic reagent [53, 54]. In the dual-wave *β*-correction spectrophotometric technique, quantification of the correct absorbance equivalent to the fractions of the chromogenic reagent that reacted with the analyte in the presence of excess of the chromogenic reagent is very much possible for precise analysis of the analyte [52–54]. *β*-Correction approach offers simple, rapid, cost-effectiveness, and selective over most of the accessible modern instrumentation and also improves the sensitivity, precision, and accuracy of the ordinary single-wavelength spectrophotometry by solving the problem arising from the interference due to the excess chromogenic reagent [54–58]. Thus, dual-wave *β* correction spectrophotometry is the most significant and well-defined aspect for measuring the correct (real) absorbance of the formed colored Cu^2+^-zincon complex.

Recently, speciation of copper species forms of copper is essential for analytical laboratories in the copper industry because of the technological importance of such analytical information [59, 60]. Kumar et al. [59] have reported that, ordinary natural organic compounds such as humic acid (HA), fulvic acid (FA), phenols, and surfactants can complex with copper, influencing its speciation and decreasing its bioavailability. To the best of our knowledge, zincon reagent has been used for detection of Cu^2+^ and other metal ions in water samples using only ordinary single-wavelength spectrophotometric methods [6, 15, 40–43]. Therefore, the current study was aimed to: (i) developing a low cost, and selective surfactant assisted *β*-correction spectrophotometric assay for total determination and copper speciation in water using zincon (ESI.  1) and (ii) assigning the stoichiometry, stability, and thermodynamic behavior of Cu^2+^ -ZI chelate. A cohesive collaboration of industry and academic institutes will be desired to miniaturize and automate the developed assay, where it has the advantages of miniaturization, automation, simplicity, and sensitivity.

## 2. Experimental

### 2.1. Apparatus

The electronic spectra and absorbance of the reagent and its Cu^2+^-zincon complex were recorded using UV-Vis spectrophotometer (Shimadzu UV-Vis 1800, Japan) connected to a Shimadzu TCC-ZUOA temperature controller unit. A Perkin-Elmer ICP MS (Sciex model Elan DRC II, USA) was also employed as a standard technique for copper analysis at the optimized operational parameters summarized in ESI. 2. A Perkin Mattson 5000 FTIR spectrometer was used for recording the FTIR. A Volac digital micropipette (10–100 *μ*L) and a Jenway pH-meter (model 3510) were used for preparation of diluted solutions in deionized water and pH measurements, respectively.

### 2.2. Chemicals and Reagents

Analytical reagent (A.R.) grade chemicals and reagents were used as received. All laboratory glassware's including high-density polyethylene (HDPE) bottles were soaked in hot detergent, soaked in HCl solution (50% v/v)-conc. HNO_3_ (2.0 M) at 1 : 1 v/v ratio rinsed with deionized water and finally dried at 80°C in an oven. Sodium salts of humic acid, fulvic acid, and phenol HA were purchased from Sigma-Aldrich. HDPE sample bottles were soaked overnight, washed with HNO_3_ (10%, v/v) solution, and rinsed with deionized water prior to use, and finally placed in precleaned HDPE. A stock solution of Cu^2+^ (1 mg/mL) was prepared by dissolving the appropriate mass of Cu (NO_3_)_2_. 3H_2_O (BDH, Poole, England) chemicals) in Milli-Q water. Zincon reagent and other metal salts were purchased from Shanghai Macklin Biochemical Co., Ltd. (Shanghai, China). More diluted standard Cu^2+^solutions were prepared by dilution with deionized water. A stock solution of zincon (1.0 × 10^−3^M) was prepared in minimal amount of ethanol, and the solution was completed to the mark with Milli-Q water. Stock solutions (1000 *μ*g/mL) of the salts AgNO_3_, MgCl_2_, BaCl_2_, CaCl_2_, CdCl_2_, Co (NO_3_)_2_, Fe_2_ (SO_4_)_3_, HgCl_2_, KCl, MnSO_4_, KMnO_4_, NiSO_4_, Pb (NO_3_)_2_, ZnSO_4_, NaCl^−^, and KIO_3_ were prepared individually by dissolving the required weights of their salts in Milli-Q water. More diluted concentrations of these salts were also prepared by proper dilution with deionized water to give varying concentrations of diverse ion. A series of Britton–Robinson (B-R) buffer of pH 2–11) was prepared as reported [61]. The surfactants benzyldimethyltetradecylammonium chloride (BTAC), sodium dodecyl sulfate (SDS), and Triton X-114 (T-X 114) were purchased from Sigma (Oregon, MO, US). Stock solutions (100 *μ*g/mL) of the surfactants were prepared individually in water as model “surfactants.” Millipore water (18.25 MΩ cm^−1^) was provided from the Milli-Q Plus system (Millipore, Bedford, MA, USA) in all experiments. Stock and more diluted Cu^2+^, HCl (0.1 M), and NaOH (0.1 M) solutions were prepared from deionized water.

### 2.3. Recommended Procedures for Cu^2+^ Assay

A series of measuring flasks (10 mL) containing standard concentrations (0.05–2.4 *μ*gmL^−1^) of Cu^2+^ ions was mixed with an excess known concentration (5.0 × 10^−5^ M) of zincon in the presence of BR buffer of pH 3. The solution mixtures were completed to the mark with Milli-Q water in the presence and absence of SDS (10 *μ*g mL^−1^). The reaction mixtures were allowed to stand for 3–5 min at room temperature. The absorbance of the solutions was measured at 465 nm (*λ*_1_) and 625 nm (*λ*_2_) against reagent blank. The true absorbance (*A*_*c*_) of the Cu^2+^-zincon in the test solutions was then computed successfully for the construction of the calibration plot.

### 2.4. Analytical Applications

#### 2.4.1. Determination of Cu^2+^ in Water Samples

In dark precleaned glass HDPE bottles glass, tap water samples (200 mL) were collected as real samples of Cu^2+^ ions from the domestic tap water, which was left to run for 20–25 min prior to sampling at the Chemistry Laboratory of the Center of the Excellence in the Environmental Studies, King Abdulaziz University, Jeddah city, Saudi Arabia. The samples were spiked with known concentrations of Cu^2+^(0.4–2.4 *μ*g mL^−1^) and instantly filtered through a 0.45 *μ*m pore-size cellulose membrane filter (Millipore Corporation) to remove all suspended particles and stored in HDPE sample bottles at 4°C prior to analysis. Cu^2+^ ions in the sample solutions were determined from the linear calibration plot of the developed dual-wave *β*-correction procedures using the following equation:

(1) $$\begin{array}{c} \left[{\text{Cu}}^{2+}\right]=b\frac{C_{\text{std}}}{m.}V_{x}, \end{array}$$

where *b* and *m* are the intercept and slope of the linear calibration plot, respectively, *C*_std_ is the known Cu^2+^ concentration, and *V*_*x*_ is the sample volume. Alternatively, the standard addition method was performed as follows: The corrected absorbance was computed in the absence and in the presence of known fractions (20.0–100 *μ*L) of the standard Cu^2+^ under the optimized parameters. The corrected absorbance (*A*_*c*_) of each solution was subsequently evaluated, and the Cu^2+^ content was then calculated via the extrapolated abscissa of the linear plot of the standard addition, employing the following equation:

(2) $$\begin{array}{c} \left[{\text{Cu}}^{2+}\right]=C_{\text{std}}\times \frac{{\text{Ac}}_{\text{sam}.}}{{\text{Ac}}_{\text{std}.}}, \end{array}$$

where *C*_std_ is the known Cu^2+^ concentration, Ac_sam_ and Ac_std_ are the real absorbance exhibited by the unknown and after adding known Cu^2+^ concentrations, respectively.

#### 2.4.2. Total Determination and Speciation of Cu^2+^ in Water

An approximate volume (0.3–0.5 L) of the tap water (TW) and mineral water (MW) samples were collected in HDPE bottles, filtered through a 0.45 *μ*m pore size cellulose membrane filter, and stored in precleaned HDPE sample bottles (0.5 L) at 4°C. Known volumes (5.0 mL) of the water sample adapted to pH 3 were transferred to measuring flasks (25.0 mL) containing zincon and standard Cu^2+^ concentrations at the optimized conditions. The solutions mixtures were completed to the mark with Milli-Q water, and the absorbance at *λ*_1_ and *λ*_2_ was recorded. The corrected absorbance's were calculated before and after spiking of standard Cu^2+^ concentrations from the standard addition linear plot. Another water sample was exposed to UV radiation at 254 nm for 4 h in the HCl (10% v/v), stored in HDPE bottles, and subjected to Cu^2+^ analysis within one day of collection as follows: Transfer an accurate volume (5.0 mL) of prefiltered water sample onto a series of measuring flasks containing zincon at the optimal parameters. Based on these bases, the Ac of the first aliquot (Ac_1_) will be a measure of labile Cu^2+^ ions in the mixture, while the Ac of the second aliquot (Ac_2_) is a measure of the sum of labile and chelated Cu^2+^ with organic matter in the aliquot. The difference of corrected absorbance (Ac_2_-Ac_1_) is a measure of the complexed fractions of Cu^2+^ in water samples.

## 3. Results and Discussion

### 3.1. Electronic Spectra of Zincon and Its Cu^2+^-Chelate Omitted

**Figure 1:**
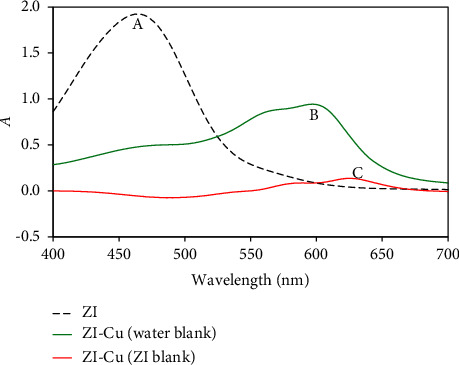
Absorption UV-visible spectra of zincon (1.0 × 10^−4^ (M) (reference water) (A); Cu^2+^-ZI complex (47.62 *μ*M) (reference water) (B) and Cu^2+^-ZI complex (reference zincon reagent) (C).

Zincon reagent (ESI.  1) contains four protonating groups: two acidic, sulfonic (pKa1) and carboxylic (pKa2); and two basic, a secondary amine (pKa3) and a phenolic one (pKa4). The most acidic group of zincon is the sulfonic group, which is usually omitted since zincon is commercially available as a monosodium salt and because of its rapid decomposition in acidic pH [16, 49]. Spectrophotometric measurements of zincon display significant change of the absorption at 565 nm around pH 4, which is characteristic for the carboxylic group rather than the sulfonic one. The UV-visible spectrum of zincon vs. water displayed distinct peak at 465 nm (*λ*_1_) and was safely assigned to *n*⟶*π*^*∗*^ electronic transition (Figure 1, curve A) [62, 63]. The UV-visible spectrum of the reaction product of zincon with Cu^2+^ at pH = 3 (Figure 1 curve B) revealed broad and ill-defined peak like shoulder in the range 563–569 nm and a strong absorption peak at 600 nm. These peaks were safely assigned to charge transfer (L⟶MCT) and electronic *d*⟶*d* transitions from shorter to longer wavelength in tetrahedral environment, respectively 16, [63]. The spectrum of Cu^2+^-zincon chelate vs. zincon (Figure 1, curve C) displayed strong peak at 625 nm (*λ*_2_). The experiential bathochromic (red) shift and the high value of the molar absorptivity (*ε* = 2.5 × 10^4^ L M^−1^ cm^−1^) suggested the suitability of the produced colored Cu^2+^-zincon chelate for establishing simple, cost-effectiveness, and reliable *β*-correction spectrophotometry approach for Cu^2+^ detection and speciation of Cu^2+^ species in water.

In the aqueous reaction media, the interference popping up from extra zincon reagent can be eliminated and the true (corrected) absorbance (Ac) of the formed Cu^2+^-zincon chelate can be computed employing the equation [53–59]:

(3) $$\begin{array}{c} \text{Ac}=\frac{∆A-∆A^{\prime }}{1-\alpha \beta }, \end{array}$$

where ∆*A* is the absorbance of the Cu^2+^-zincon complex at *λ*_2_, whereas ∆*A*′ is its absorbance at *λ*_1_ versus zincon (reference blank). The spectral parameters *α* and *ß* were computed using the following equations [56, 57]:

(4) $$\begin{array}{c} \beta =\frac{A_{0}}{A_{0}^{\prime }}=\frac{\varepsilon_{L}^{\lambda 2}}{\varepsilon_{L}^{\lambda 1}}, \end{array}$$

(5) $$\begin{array}{c} \alpha =\frac{A_{\alpha }^{\prime }}{A_{\alpha }}=\frac{\varepsilon_{\text{ML}}^{\lambda 1}}{\varepsilon_{\text{ML}}^{\lambda 2}}, \end{array}$$

where *A*_*α*_ is the absorbance of the produced Cu^2+^-zincon chelate in solution at *λ*_2_ while *A*_*α*_′ is it's absorbance at *λ*_1_ versus water (blank). *A*_0_′ and *A*_o_ are the absorbance of the blank solution at *λ*_1_ and *λ*_2_, against water, respectively. Thus, the planned *β*-correction spectrophotometry assay offered good sensitivity over ordinary spectrophotometry by suitable choice of *λ*_1_ and *λ*_2_ at the sink and the peak of the visible spectrum of Cu^2+^-zincon chelate versus reagent blank [53–59], respectively. The lowest and the highest absorbance of Cu^2+^-zincon chelate at *λ*_1_ (465 nm) and *λ*_2_ (625 nm) versus zincon (Figure 1, curve C) was used for calculating the corrected absorbance. Employing single wavelength spectrophotometry, the absorbance of the developed Cu^2+^-zincon chelate at *λ*_2_ versus zincon was lower than the corrected absorbance (Ac) computed via dual-wave *β*-correction spectrophotometry [53, 54] in consistence with the data reported [58, 59]. The value of *β* computed from Figure 1 (curve A) and equation (4) was 0.18, whereas the value of *α* calculated from Figure 1 (curve B) and equation (5) was 2.18 in close consistence with the data published [53–59].

### 3.2. Programing of the Analytical Parameters

**Figure 2:**
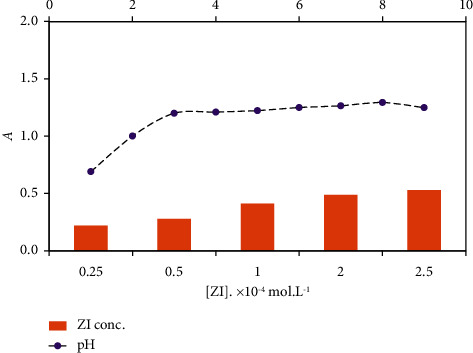
Plots of the corrected absorbance of Cu^2+^ (47.62 *μ*M) versus solution pH and zincon concentrations (2.5 × 10^−5^-2.5 × 10^−4^ (M) at 625 nm at room temperature.

To explore the impact of pH (pH 1.0–10.0) of the aqueous solution on the developed Cu^2+^-zincon colored chelate, the electronic spectra and the actual absorbance of Cu^2+^ (3.0 *μ*gmL^−1^) solution containing zincon (1.0 × 10^−4^ M) were recorded at various pH. The absorbance of the produced colored Cu^2+^-zincon complex reached its maximum value at pH 3.0 (Figure 2(a), dotted line). The binding sites of phenolic OH and azo (–N=N-) groups of zincon reagent at pH 3 are capable to coordinate with Cu^2+^ [54] in consistence with the data published for the Cu^2+^-zincon complex [54, 55]. Therefore, the solution pH was adopted at pH 3 in the following study.

An aqueous solution of Cu^2+^ (3.0 *μ*g mL^−1^) at pH 3 was allowed to react with various known concentrations of zincon (2.5 × 10^−5^-2.5 × 10^−4^ M). The results are demonstrated in Figure 2(a) (column), where zincon concentration of 1.0 × 10^−4^ M was found enough for detection of Cu^2+^ up to 5.0 *μ*g mL^−1^ in the solution. The calculated molar ratio of ligand to Cu^2+^ was found 2 : 1 suggesting formation of Cu^2+^-ZI complex of the formula Cu (L)_2_, where L = ZI (ESI.  1).

**Figure 3:**
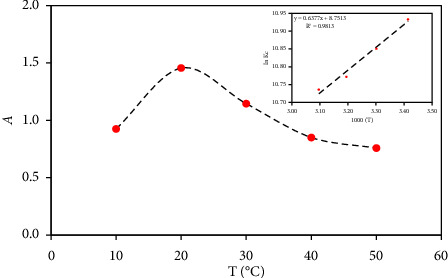
Plots of the corrected absorbance of Cu^2+^ (47.62 *μ*M)-zincon complex versus solution temperature (10–50°C) at 625 nm at pH 3. Inset plot of ln *K*_*c*_ vs. 1000/T (K^−1^) of the Cu^2+^-ZI complex. Conditions: [Cu^2+^] = 47.62 *μ*M and [ZI] = 1.0 × 10^−4^ M.

The impact of the solution temperature (10–50°C) on the absorbance of the Cu^2+^-zincon {3.0 *μ*g mL^−1^ Cu^2+^} at pH 3 was examined. The data are displayed in Figure 3. The absorbance of the formed Cu^2+^-zincon chelate increased on growing temperature up to 25°C, followed by a gradual decrease in the absorbance. The degradation of the formed Cu^2+^-zincon chelate and/or the decrease in the interaction between Cu^2+^ ions and ZI at a temperature higher than 25°C are most likely accounted for the observed trend. Hence, a room temperature (25 ± 1°C) was selected as a suitable condition for the formation of the Cu^2+^-ZI complex.

The absorbance of Cu^2+^-ZI at known Cu^2+^ (2.0 *µ*g mL^−1^) and zincon (1.0 × 10^−4^ M) concentrations after mixing was recorded immediately over a wide range of time (0.0–80 min at pH 3. The absorbance and the corrected absorbance (*A*_*c*_) of the produced Cu^2+^-ZI complex were measured at several shaking time intervals (0.0–85 min) employing single wavelength and *β*-correction spectrophotometry, respectively. The colored Cu^2+^-ZI complex was established within 1-2 min of shaking, and the absorbance remained constant up to a standing time of 85 min (ESI.  3). These data added further provision to the analytical utility of the established Cu^2+^-ZI complex for developing a solvent-free *ß*-correction spectrophotometry assay for Cu^2+^ detection in water. Thus, in the next study, the absorbance of the Cu^2+^-Zi complex was measured within 80 min of mixing at pH 3.

**Figure 4:**
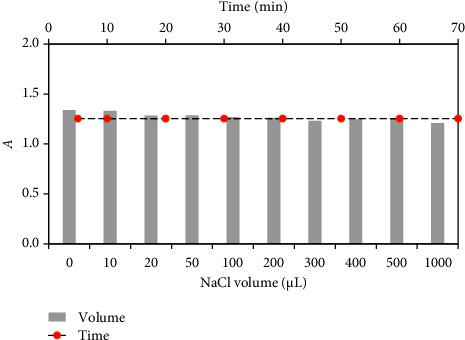
Influence of standing time (0.0–70 min) and various volumes (0.0–1000 *μ*L) of NaCl concentration (1.0 × 10^3^ *μ*g mL^−1^) on the absorbance of Cu-ZI complex. Conditions: [Cu^2+^] = 47.62 *μ*M, [ZI] = 1.0 × 10^−4^ M and solution pH 3 at 625 nm.

The influence of various proportions (0.0–1000 *µ*L) of NaCl (1.0 × 10^3^ *µ*gmL^−1^) and standing time (0.0–70 min) on the absorbance of the tested Cu^2+^-Zincon complex at pH 3 under the optimal parameters was also studied. The plots of the absorbance of the formed Cu^2+^-ZI complex in the presence of various volumes of the salt added and time are shown in Figure 4. In the absence of the salt added, the corrected absorbance of the Cu-ZI complex computed for the added salt concentration was about 4.2% (Figure 4). These results, clearly simplifies that Cu^2+^ complexation with ZI was less influenced by the salt added to the medium. Thus, in the next study, no salt was added to the reaction medium.

### 3.3. Thermodynamic Parameters of Cu^2+^-ZI Complex

The thermodynamic features of the developed Cu^2+^-ZI complex in the temperature range of 293–323 K were determined. The Cu^2+^ species present as neutral species at pH 3 only one species of Cu^2+^-ZI chelate existed, and no precipitation obtained. On rising the solution temperature from 293 to 323 K, the equilibrium constant (*K*_*c*_) decreased signifying that, the complex formation is exothermic process [64, 65]. The slope and intercept of the linear plot of ln *K*_*C*_ versus 1000/T (Figure 3, inset) were used for calculating the Δ*H*, Δ*S*, and Δ*G* of the formed Cu^2+^-ZI complex. The Δ*H*, Δ*S*, and Δ*G* were found −5.3 kJmol^−1^, 72.75 J mol^−1^ K^−1^, and −26.97 kJ mol^−1^ (at 298 K), respectively. The value of the Δ*H* (−5.3 kJ mol^−1^) reveals the exothermic nature and the bond energy difference between zincon and its Cu^2+^ complex. Growing temperature minimizes Cu^2+^ interaction with ZI, resulting in decrease in the percent yield of the complex. The Δ*G* value (−26.98 kJ mol^−1^) at 298 K decreased on increasing temperature, supporting the spontaneous nature of the complex.

### 3.4. Selectivity Study

#### 3.4.1. Impact of Diverse Ions

The analytical utility of the established *β*-correction spectrophotometry for Cu^2+^ (3.0 *μ*g mL^−1^ (47.62 *µ*M) in the presence of excess of various potentially interfering major ions with Cu^2+^ at concentrations representative of many fresh waters, tap, and mineral water at the optimal pH 3 and ZI (1.0 × 10^−4^M) is critical. Thus, the interference of of ions Cl^−^, Br^−^, OH^−^, NO_3_^−^, SO_4_^2−^, and metal ions, e.g., Na^+^, K^+^, Cd^2+^, Ni^2+,^ Sr^2+^, Mn^2+^, Fe^3+^, Co^2+^, Pb^2+^, Hg^2+^, and Ag^+^, and the oxy ions (MnO_4_^−^, IO_3_^−^, and WO_4_^2−^) was studied individually in the presence of Cu^2+^-ZI complex at the optimized parameters of pH and zincon concentrations. The absorbance of Cu^2+^-ZI complex was compared with that in the presence of the interfering species. The tolerance limit (acceptance edge) (m/m) was distinct as the added concentration of the interfering species producing a relative standard deviation (RSD) of ±5% of the true absorbance of Cu^2+^-ZI chelate. The results obtained are summarized in Table 1. The ions Cl^−^, Br^−^, NO_3_^−^, SO_4_^2−^, IO_3_^−^, Na^+^, K, and Ca^2+^ revealed negligible change in the corrected absorbance of Cu^2+^-ZI complex at 1 : 1000 molar excess of Cu^2+^ to the diverse species. The ions Cd^2+^, Ni^2+^, Sr^2+^, Mn^2+^, Cr^3+^, Hg^2+^, and Pb^2+^ were tolerable up to 50-fold excess to Cu^2+^. The ions Cr^3+^, Hg^2+^, and Pb^2+^ at level up to 20 fold greater than Cu^2+^ ions did not interfered on the absorbance of Cu^2+^-zincon chelate, whereas Fe^3+^, Co^2+^, and Ag^+^ ions interfered extremely with the complex. The interference of Fe^3+^ and Co^2+^ was masked by adding a few drops of NaF (0.1% m/v) via the formation of colorless [FeF_6_]^3−^ complex, whereas Co^2+^ interference was minimized by adding ethanolamine (0.01%) to form a colorless complex in the test aqueous solution. The oxyanions MnO_4_^−^ and WO_4_^2−^ were tolerable by adding a few drops of sodium azide (NaN_3_).

#### 3.4.2. Effect of Surfactants

**Figure 5:**
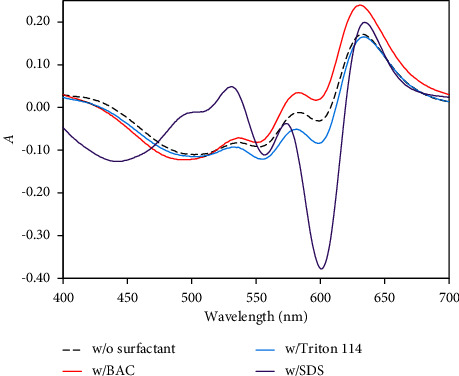
Absorption UV-visible spectra of Cu^2+^-ZI chelate at zero and after addition of known concentrations (10.0 *μ*gmL^−1^) of SDS, BTAC, and Triton114 individually. Condition: [Cu^2+^] = 4.7 *μ*M, [ZI] = 1.0 × 10^−4^ M and solution pH 3 at 625 nm.

The impact of surface-active agents (0.1–10 ppm) on the stability and the corrected absorbance of Cu^2+^-zincon chelate in the developed procedure are critical. Thus, the impact of all kinds of surfactant such as cationic (BTAC), anionic (SDS) and nonionic (Triton X-100) on the selectivity of the established assay for Cu^2+^ was studied at the optimized condition. Cationic and nonionic surfactants have nonsignificant changes on the absorbance of the developed colored Cu^2+^-ZI chelate, whereas in the presence of SDS, a synergistic increase in the value of the absorbance was only seen (Figure 5). The impact of SDS may be due to its ability to form versatile interactions including electrostatic, hydrophobic, bi-bi interaction, complex ion association, and/or H bonding with the Cu^2+^ complex. The change in the effective microenvironment by SDS micellar solution around Cu^2+^ in the aqueous solution and their contribution to the physicochemical features such as rate constant and spectral characteristics may also participated in the trend observed. The possible association between the cationic Cu^2+^-zincon complex and SDS as a bulky anion by forming ternary complex ion associate between SDS may also enhanced the molar absorptivity of the produced ternary complex ion associate. The available hydroxy groups and water molecules may also screened by SDS at the boundary and subsequently resulting in a worthy association between ZI and Cu^2+^ ions. Thus, it is worthy to note that, the use of SDS is attractive in developing surfactant assisted *β*-correction spectrophotometry for Cu^2+^ detection owing to its low cost, toxicity, and reduced environmental impact. Thus, the effect of SDS in the absorbance was continual to improve the detection of Cu^2+^.

### 3.5. Stoichiometry and Stability of Cu^2+^-ZI Complex

The stoichiometry and stability of the formed Cu^2+^-ZI chelate are critical in testing the analytical utility of the colored Cu^2+^-zincon chelate for Cu^2+^ detection [59–62]. The impact of standing time (0.0–100 min) on absorbance of the Cu^2+^-ZI chelate at 625 nm using Cu^2+^ (3.0 *μ*gmL^−1^) and 1.0 × 10^−4^ M ZI at pH 3 was recorded (ESI.  3). The data revealed good stability over a period up to 100 min, revealing the good stability of the produced copper (II) chelate. Assuming the existence of one complex species of Cu^2+^-ZI, Job's and mole ratio methods at pH 3 [62, 63] were used to determine the stoichiometry and stability of the formed Cu^2+^-ZI chelate. The results of Job's (ESI.  4) suggested formation of 1 : 2 stoichiometry of Cu^2+^-zincon complex. The stoichiometry of Cu^2+^: zincon was also supported from the mole ratio plot (ESI.  5) [62]. Reflectance electronic spectrum of Cu^2+^-zincon complex also displayed broad peak cantered at 17241cm^−1^ interpretable in terms of square planar stereochemistry [16, 66, 67]. The FTIR spectra of the free zincon reagent and its copper (II) chelate are displayed in ESI. 6. The v(C=N) band in the complex was found in the same position of the FTIR of free zincon indicating no participation of the azomethine in the complex formation with Cu^2+^ ions [16, 66]. The spectra added further support to the participation of the azo (-N=N-) group N and the involvement of the phenolic OH in the complex formation with Cu^2+^ ions (see ESI.  6) [66]. Thus, it can be concluded that, the zincon coordinated to Cu^2+^ via two N and two O of the azo and phenolic OH groups, and the structure of the produced copper-zincon complex can be postulated as [CuL_2_], where L = zincon and the most probable structure of Cu^2+^–zincon chelate, can be proposed as shown in ESI.  1.

The stability constant (K) of the Cu^2+^-ZI complex was further successfully computed from Job's plot (ESI.  4), where the extrapolated absorbance (*A*_extp._) near to the equivalence point corresponds to the absorbance of the Cu^2+^-ZI complex. Based on the Job's plot (ESI.  4), the Cu^2+^-ZI complex is dissociated in the area of extrapolation, and the true absorbance of the produced Cu^2+^ complex is to some extent lower than the hypothetical value. Thus, the produced Cu^2+^-ZI complex can be stated by the following chemical equilibrium [62]:

(6) $$\begin{array}{c} {\text{Cu}}^{2+}+2L^{-}⇌\left[{\text{CuL}}_{2}\right], \end{array}$$

where L = zincon ligand (L) in the complex formation, and [CuL_2_] refer to the formed Cu^2+^ chelate, respectively. The fraction of the true corrected absorbance (Ac) at a given value on the *X*-axis of Job's plot (ESI.  4) to the extrapolated absorbance (*A*_exp_) determined from the extrapolated lines corresponding to the same point in the *X*-axis is equivalent to the mole fraction of the formed chelate [CuL_2_]. The *K*_*s*_ value of the formed [CuL_2_] complex was also computed from Job's plot (ESI 4) using the following equation (7) [62]:

(7) $$\begin{array}{c} K=\frac{\left(A/A_{\text{expt}}\right).C}{\left.\left(C_{m}-\left(A/A_{\text{expt}}\right).C\right)\left[C_{x}-\right.\left(A/A_{\text{expt}}\right).C\right)}, \end{array}$$

where *C*_*m*_ is the molar analytical concentration of the metal, *C*_*x*_ is the total molar analytical concentration of the ligand depending on the controlling reactant at the end point and *n* is ligand to metal ratio in the Cu^2+^-zincon chelate, respectively. The average computed *K*_*s*_ value (2.0 × 10^6^) displayed acceptable stability and the suitability of the Cu^2+^-ZI complex for developing spectrophotometric method for Cu^2+^ determination.

### 3.6. Analytical Performance and Figures of Merits

**Figure 6:**
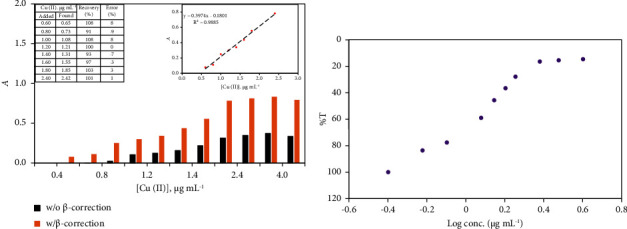
Calibration plot for Cu^2+^ detection using ordinary single wavelength and *β*-correction spectrophotometry (a). Inset calibration curve using the corrected (A) and Ringbom's plot using *β*-correction spectrophotometry of Cu^2+^-ZI complex (b). Conditions: [ZI] = 1.0 × 10^−4^ M and solution pH 3 at *λ*_max_ 625 nm.

At pH = 3 and zincon concentration (1.0 × 10^−4^ M), Beer's–Lambert plots of the absorbance versus Cu^2+^ concentration were found linear over the range 0.50–2.4 *µ*g/mL (7.9–38.1 *µ*M) and 1.6–3.0 *µ*g/mL (25.4–47.62 *µ*M) employing the developed *β*-correction and ordinary spectrophotometry, respectively. The obtainable results are illustrated in Figure 6(a). The calibration plots using single wavelength and the established *β*-correction spectrophotometry were conveyed by the following regression equations (8) and (9), respectively:

(8) $$\begin{array}{c} A=1.6\times 10^{-1}\text{ Cx}-7.5\times 10^{-3}\left(R^{2}=0.9907\right), \end{array}$$

(9) $$\begin{array}{c} \text{Ac}=3.9\times 10^{-1}\text{Cx}-1.7\times 10^{-1}\left(R^{2}=0.9567\right). \end{array}$$

The molar absorptivity (*ε*) and Sandal's sensitivity index [67, 68] computed from Beer's–Lambert plots of Cu^2+^-ZI complex were 2.5 × 10^4^ L mol^−1^ cm^−1^ and 0.003 *µ*g cm^−2^ using the *β*-correction assay and 1.0 × 10^4^ L mol^−1^ cm^−1^and 0.006 *µ*g cm^−2^ utilizing single wavelength spectrophotometry. Thus, the established *β*-correction spectrophotometric assay presented a worthwhile approach for improving the sensitivity and selectivity of Cu^2+^ in sensing over the ordinary one.

Ringbom's plot [67] of log Cu^2+^ concentration versus percent transmittance (T%) was assumed to quantify the optimal concentration range of Cu^2+^ for the system that follows Beer's–Lambert equation (Figure 6(b)). The effective linear concentration range for Cu^2+^ by the developed *β*-correction was in the range 1.1–2.4 *µ*g mL^−1^ (17.5–38.1 *µ*M). The LOD and LOQ values of Cu^2+^ [68] using ordinary spectrophotometry were 0.19 *μ*g mL^−1^ (3.0 *µ*m) and 0.63 *μ*g mL^−1^ (10.0 *µ*m) and significantly improved to 0.08 *μ*g mL^−1^ (1.27 *µ*m) and 0.26 *μ*g mL^−1^ (4.13 *µ*M) Cu^2+^ ions employing the established dual-wavelength *β*-correction spectrophotometry, respectively. These results reflected the significant improvement of the sensitivity by about 50%. The linear dynamic range (LDR) for the *β*-correction spectrophotometry embedded in Figure 6(a) (right inset) is higher than that corresponding to the uncorrected absorbance. The LOD of the established assay based on corrected absorbance (Ac) was significantly improved. The *β*-correction assay exhibited better recoveries (91–108%) with a relative standard deviation (RSD) in the range 3–5% lower than the ordinary single wavelength method. The *β*-correction assay has also displayed great advantages in improving the peak shapes and reducing matrix effects *compared* to ordinary spectrophotometry (Figure 6(a)). The established assay took the advantage of the reduced matrix effects without complex preprocessing of the samples, greatly simplifying the experimental process on comparing with the existing methods for Cu^2+^ (Table 1) [41–51].

Further, the LOD and LOQ of the surfactant- (SDS-) assisted *β*-correction spectrophotometry were enhanced from 0.08 *μ*g mL^−1^ (1.27 *µ*M) and 0.26 *μ*g mL^−1^ (4.13 *µ*M) to 0.02 *μ*g mL^−1^ (0.32 *µ*M) and 0.078 *µ*g/mL (1.24 *µ*M), respectively. The change in the effective microenvironment by micelles solution around Cu^2+^ ions may also improve formation of ternary ion associate of Cu^2+^-zincon complex and SDS. The hydroxy groups and the available free water molecules may also screened by SDS at the boundary and successively resulting in better organization between ZI and Cu^2+^ions.

The precision and accuracy of the proposed *β*-correction spectrophotometry assay were further computed from the recovery of three replicate measurements of known Cu^2+^ concentrations in water. The results demonstrated in Figures 6(a) (Table inset) revealed good performance of the planned *β*-correction spectrophotometric assay and support the current protocol for Cu^2+^ detection in water. Further, a judgment between the efficacies of the established *β*-correction spectrophotometry with several reported spectrometric methods is summarized in Table 2. The LOD, LOQ, and LDR of developed surfactant assisted-correction spectrophotometry assay are favorably associated with most of the established spectrophotometric protocols (Table 2). The LOD of the planned *β*-correction assay was higher than the LOD (0.018 *μ*g L^−1^) using paper-based chip for fluorescence Cu^2+^ detection (Table 2). However, the measured value by the established assay below the acceptable limit of Cu^2+^ fixed by WHO and US-EPA in water. The proposed assay frees from the interfering of various anions and cations present in water samples. Thus, the proposed assay could be suitable for Cu^2+^ detection in water and it has the benefits of low cost, simple, practical, and eco-friendly.

### 3.7. Analytical Applications and Validation of the Established Methodology

**Figure 7:**
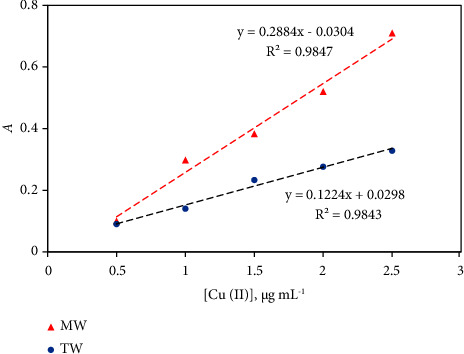
Plot of the standard addition for Cu^2+^ detection as Cu^2+^-ZI complex in mineral (MW) and in tap (TW) water samples.

Due to the unavailability of certified reference materials (CRM) for Cu^2+^ to check the reliability and validity of the established assay for Cu^2+^ detection, known concentrations (0.4–2.4 *μ*gmL^−1^) of Cu^2+^ were spiked into mineral water (MW) and tap water (TW) samples as mentioned before and analyzed using the established *β*-correction spectrophotometric assay. The results of Cu^2+^ determined in tap (TW) and mineral (MW) water samples are summarized in Table 3. Representative plots for measuring Cu^2+^ in tap water and mineral (MW) water samples are also shown in Figure 7. The results were further validated by determination of Cu^2+^ by the official ICP-OES at the optimal operation parameters. Acceptable percentage recoveries of Cu^2+^ in tap (90.4–113.9%) and mineral (97.5–110.7%) water samples were attained. The “added,” “found” and recovery percentage (91–102%) of Cu^2+^ concentrations were found comparable and acceptable. At 95% confidence (*n* = 5, *P* < 0.05) [66], the tabulated Student *t*_tab_ and *F* values were greater than the experimental Student *t*_exp_ (1.96–2.1) and *F*_exp_ (2.3–2.8), respectively, revealing acceptable consequence between the tabulated and experimental values for the detection of Cu^2+^ in water.

## 4. Conclusion, Advantages, Limitations, and Outlooks

In summary, our research demonstrates the potential for the development of significant surfactant simple, effective, cost-effectiveness, interference, and solvent free *β*-correction spectrophotometric approach for total determination and speciation (labile and chelated) of Cu^2+^. The molar absorptivity of the assay reveals good sensitivity and linear range, LOD, reliability, rapid analysis, no extra costly material necessary, and free from interference of common metal ions. The planned methodology may also substitute the common analytical methods (AAS and ICP-OES) that suffered from time consuming, complicated instrumentation and multiple preconcentration steps. Further, the developed assay can be drawn-out for detection and speciation of Cu^2+^ at ultra-trace low levels in water via on-line enrichment from water samples by dispersive liquid-liquid microextraction [69] and/or on nano-sized sorbent packed column and succeeding elution prior analysis [70]. Thus, the assay can set the trend for coupling sorbent packed column that can assist as a new dimension in *β*-correction spectroscopy. The accuracy and applicability of the proposed assay were proved by recovery studies for water samples and the results were close to 100%. The absence of the interactive effects of the analytical parameters using one issue at a time represent the main drawback and might decrease the analytical utility of the current study. Accepting the positive impact of SDS in the absorbance will be studied properly in more detail to advance Cu^2+^ detection and to assign the most probable mechanism. The method could also be extended for detection of Cu^2+^ ions in natural waters with high complexing capacity of organic matter, e.g., humic, fulvic acids, phenols, surfactants, etc. Design experiment is also extremely suggested for advance the present approach for attaining effective and perfect Cu^2+^ detection.

## Figures and Tables

**Table 1:** Selectivity data and tolerance limit of diverse ions in Cu^2+^ determination by the developed dual-wave *β*-correction spectrophotometry.

Diverse ions	Tolerance limit *μ*g mL^−1^(*μ*M)
Cl^−^, Br^−^, NO_3_^−^, SO_4_^2−^, IO_3_^−^, Ca^2+^, Na^+^, K^+^	1000 (15873.02)
Cd^2+^, Ni^2+^, Sr^2+^, Mn^2+^, Cr^3+^, Hg^2+^, Pb^2^	50 (793.65)
Fe^3+^, Co^2+^, Ag^+^	20 (317.46)
MnO_4_^−^, WO_4_^2−^	100 (1587.3)

**Table 2:** A comparison between the figures of merits (*μ*M) of the developed *β*-correction and some of the reported spectrophotometric methods for Cu^2+^ detection^*∗*^.

Matrix	LDR (*μ*M)	LOD (*μ*M)	Remarks	Ref.
Vegetables and tea	0.0–16.25	0.111	Accurate, reliable, and time consuming	[41]
Soil and vegetables	2.54–20.63	0.841	pH range 8–10, interference from polyhydroxy species	[44]
	6.98–16.67	2.333		
Synthetic mixture and water	16.19–128.89	0.508	Required heating and time consuming	[45]
Natural water and pharmaceutical samples	1.59–158.73	0.159	High molar absorptivity and tolerant to many foreign species	[46]
Alloy, pharmaceutical, fertilizer, and environmental samples	Up to 277.78	6.587	Use hazard organic solvents	[47]
Food, leafy vegetables, and fertilizers environmental samples	Up to 277.78	9.524	Efficient and selective	[48]
Pharmaceutical and alloy samples	75.40–256.03	—	Long time of equilibration	[50]
Binary, synthetic mixtures, and various real samples	31.75–222.22	—	Less sensitive	[51]
Water	22.22–38.10	19.048		Present study without *β*-correction
	6.35–38.10	3.016		
Water	9.52–38.10	9.524	Precise, sensitive, and short analytical time	Present work with *β*-correction
	6.35–38.10	1.270		

^*∗*^Spect = spectrophotometry; LDR = linear dynamic range, LOD = limit of detection.

**Table 3:** Analytical results of Cu^2+^ determination in tap and mineral water samples by the developed dual-wave *β*-correction spectrophotometric method^*∗*^.

Samples	Added (*μ*M)	Found (*μ*M)	Recovery (%)	Error (%)
Tap water	0.00	3.71	—	—
	7.94	7.17	90.4	−9.6
	15.87	18.08	113.9	+13.9
	23.81	22.75	95.6	−4.4
	31.75	30.29	95.4	−4.6
	39.68	40.75	102.7	+2.7

Mineral water	0.00	−0.11	—	—
	7.94	7.81	98.4	−1.6
	15.87	14.29	90.0	−10.0
	23.81	26.35	110.7	+10.7
	31.75	31.92	100.6	+0.6
	39.68	38.67	97.5	−2.5

^*∗*^Average of three measurements (*n* = 3).

## Data Availability

Electronic Supplementary data are available upon request.

## Supplementary Materials

Supplementary Materials The following figures and tables will be displayed under the SI download link online as a guide for readers. ESI. 1. Chemical structures of zincon (I) and its copper (II) complex (II). ESI. 2. ICP-OES operational parameters for copper determination. ESI. 3. Effect of standing time on absorbance of the Cu^2+^-ZI complex at 625 nm using Cu^2+^ (3.0 *μ*gmL^−1^) and 1.0 × 10^−4^ M ZI at pH 3. ESI.4. The Job method of Cu^2+^-zincon chelate; Cu^2+^ and zincon 1 × 10^−3^ M. ESI.5. Mole-ratio method of Cu^2+^-zincon complex; [Cu^2+^] = 1 × 10−3 M and [zincon] = 1 × 10^−3^ M. ESI.6. FTIR spectra of zincon and its Cu^2+^ complex.
