# Supplementary material for: An Eco-Friendly, Interference, and Solvent Free Surfactant-Assisted Dual-Wavelength β-CorrectionSpectrometric Method for Total Determination and Speciation of Cu2+ Ions in Water
Source: Int J Anal Chem. 2023 Nov 4;2023:5001869. doi: 10.1155/2023/5001869 (PMC10640129; doi:10.1155/2023/5001869)
Supplement: Supplementary Materials — The following figures and tables will be displayed under the SI download link online as a guide for readers. ESI. 1. Chemical structures of zincon (I) and its copper (II) complex (II). ESI. 2. ICP-OES operational parameters for copper determination. ESI. 3. Effect of standing time on absorbance of the Cu2+-ZI complex at 625 nm using Cu2+ (3.0 μgmL−1) and 1.0 × 10^−4^ M ZI at pH 3. ESI.4. The Job method of Cu2+-zincon chelate; Cu2+ and zincon 1 × 10^−3^ M. ESI.5. Mole-ratio method of Cu2+-zincon complex; [Cu2+] = 1 × 10^−3^ M and [zincon] = 1 × 10^−3^ M. ESI.6. FTIR spectra of zincon and its Cu2+ complex. [file 5001869.f1.docx]

**Electronic Supplementary Information’s (ESI)**

**An eco-friendly, interference and solvent - free surfactant-assisted dual-wavelength β-correction spectrometric method for total determination and speciation of Cu^2+^ in water**

D. S. Al-Raimi^1, 2^, K. M. Al-Ahmary^2^, H.A. Nasef^3^, H. Alwael^1^, T. N. Abduljabbar^1‡^, L. H. Mujawar^4^, E.Y. Danish^1^, M. T. Soomro^4^, M. S. El-Shahawi^1‡*^.

***^1^****Department of Chemistry, Faculty of Science, King Abdulaziz University, P.O. Box 80203, Jeddah 21589, Saudi Arabia*

*^2^Department of Chemistry, College of Science, University of Jeddah, Jeddah, Saudi Arabia*

*^3^ Department of Basic Sciences,*

*ta Higher Institute for Engineering and Technology, Mansoura 35111, Egypt.*

*^4^.Center of Excellence in Environmental Studies, King Abdulaziz University, P.O. Box 80216, Jeddah 21589, Saudi Arabia.*


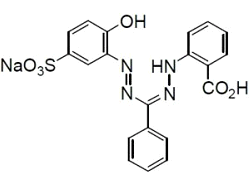


**I II**

**ESI. 1.** Chemical structures of zincon **(I)** and its copper (II) complex **(II).**

ESI. 2. ICP- OES operational parameters for copper determination.

| **Parameter** | **Unit** |
| --- | --- |
| Rf Power | 1400W |
| Nebulizer Flow | 0.7 L/min |
| Auxiliary Flow | 0.3 L/min |
| Plasma Flow | 10.0 L/min |
| Sample Pump Flow | 1 mL/min |
| Plasma Viewing | Axial |
| Processing Mode | Area |
| Replicates | 3 |
| Nebulizer Type | Cross-flow (Gim Tip) |
| Spray Chamber | Scott (Ryton) |
| Injector | Scott (Ryton) |
| wavelength | 267.7, nm |


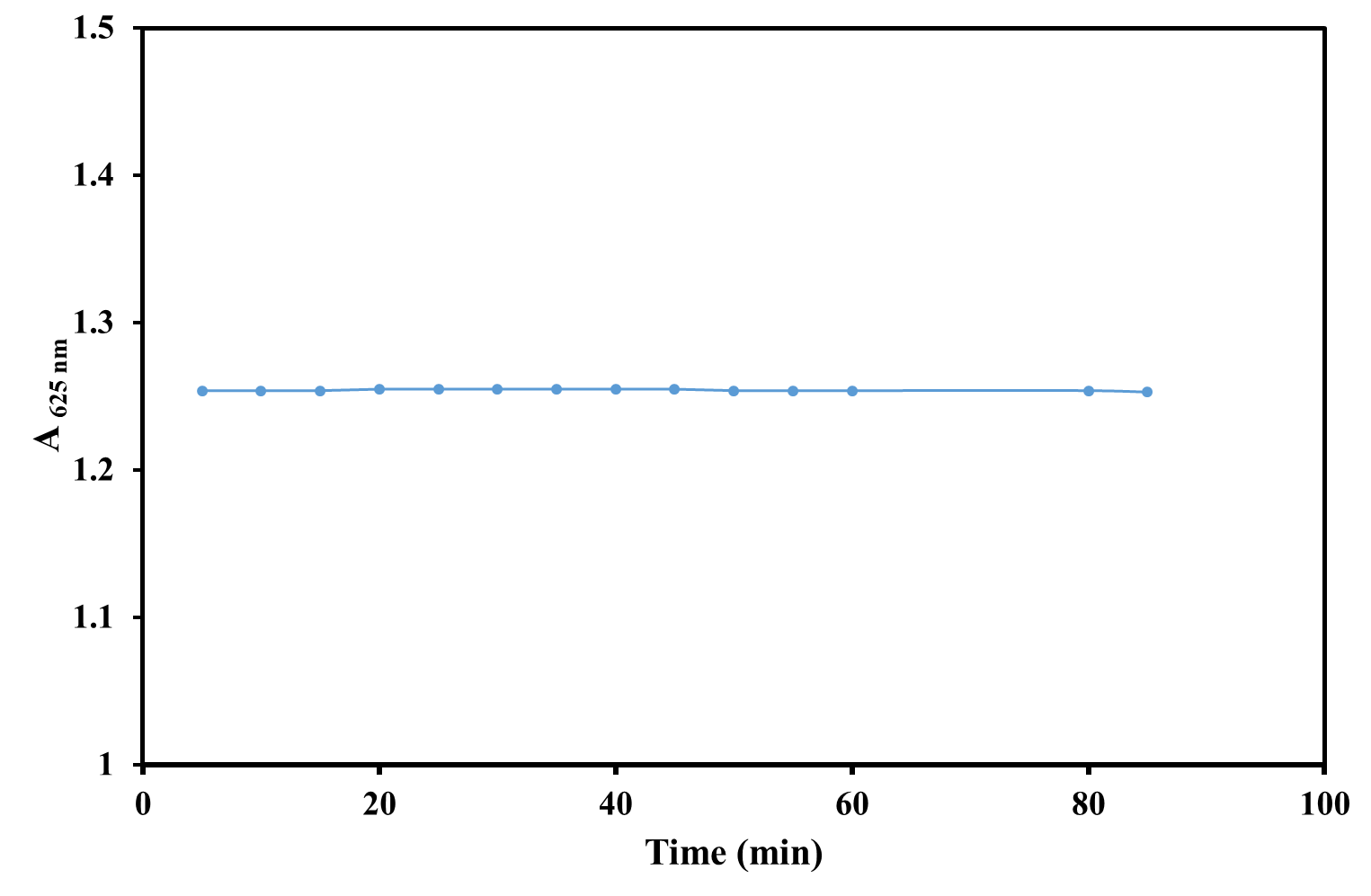


**ESI. 3.** Effect of standing time on absorbance of the Cu^2+^-ZI complex at 625 nm using Cu^2+^ (3.0 μgmL^−1^) and 1.0×10^-4^ M ZI at pH 3.

Job’s method of continuous variation (ESI. 4) [1] was employed to elucidate the composition of the complex. Equimolar solutions of Cu^2+^ and zincon (1.0×10^-4^ M) were used to determine the molar ratio of Cu^2+^ to zincon. The absorbance values were recorded at 625 nm *against* the reagent blank. The plot of the true absorbance at 625 nm *versus* mole fraction of the zincon (V_L_/V_M_+V_L_) where V_L_ and V_M_ are the volumes of the reagent and Cu^2+^ ions, respectively is demonstrated in ESI-4. The results suggested formation of 1:2 molar ratios of Cu^2+^: zincon in the Cu^2+^- zincon chelate.

**ESI.4.** Job method of Cu^2+^-zincon chelate; Cu^2+^and zincon 1×10^-3^M.

The stoichiometry of Cu^2+^: zincon was also determined from the molar ratio method [49]. The results are demonstrated in ESI.5. These results added further support of the formation of Cu^2+^-zincon of the molar ratios of 1:2 Cu^2+^: zincon and the formed complex has the molecular formula of CuL_2_ where L=zincon.


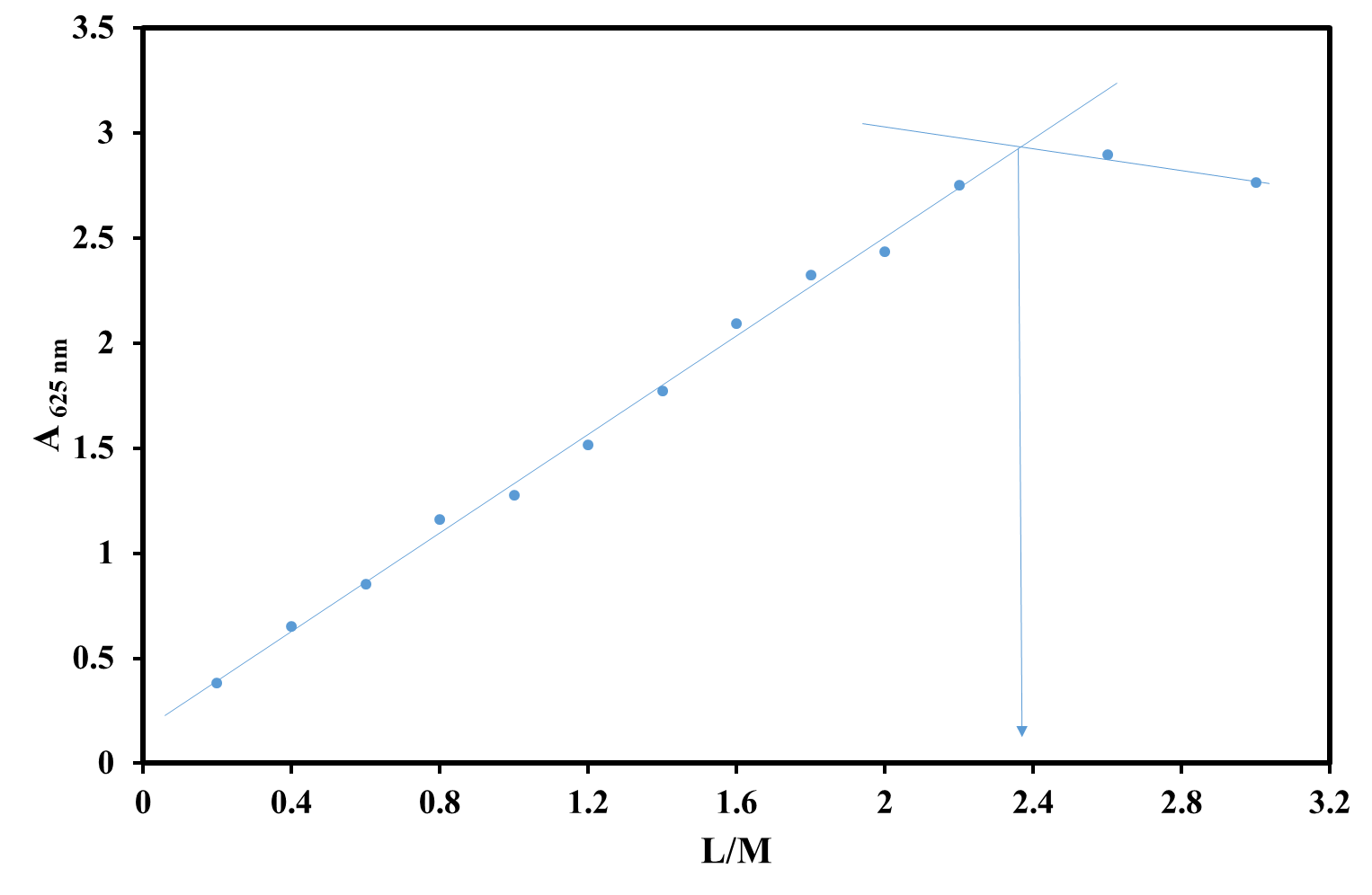


**ESI.5**. Mole- ratio method of Cu^2+^ -zincon complex; [Cu^2+^ ] = 1×10^-3^ M and [zincon] =1×10^-3 M.^

Zincon reagent (ESI. 1) has one azo nitrogen (-N=N) in addition to three ionizable sulfonic SO_3_H, COOH and phenolic OH groups. Thus, the interaction of Cu^2+^ with zincon was critically studied and the results showed considerable colour change at pH 3-4 supporting formation of Cu^2+^ -zincon complex. Zincon reagent in the test aqueous solution of pH 3-4 exists in the dissociated (anionic form) form due to the de-protonation of sulfonic acid, carboxylate and/or phenolic OH groups [2, 3]. Based on the FTIR spectra of zincon and its Cu^2+^ complex at the optimized pH 3-4 (ESI. 6), it can be concluded that, the binding sites of zincon in its complex are more likely due to the oxygen atom of phenolic OH after de protonation, and azo nitrogen (-N=N) at the used pH.

The FTIR spectra of free zincon and its copper chelate are shown in ESI. 5. The FTIR spectrum of zincon displays well defined vibrations at 1630, 3120 and 1540 cm ^-1^ and were safely assigned to v(C=N), v(N- H) and v(N= N) [2, 3], respectively. The vibrations exists at 3430 and 1680 cm^-1^ are assigned to the superimposed stretching frequencies of the phenolic OH and carboxylic OH, and the latter is attributed to v(C=O) of the carboxylic group. The vibrations at 1470 and 1320 cm ^-1^ are assigned to coupled v(C=O) and (OH) in-plane frequencies. In the IR spectrum of copper complex, the characteristic vibration of C=O was not existed and is replaced by new bands at 1550-1590 and 1440-1380 cm^– 1^ corresponding to the anti-symmetrical and symmetrical vibrations of CO group, respectively revealing no coordination of carboxylate in the complex formation with Cu^2+^ ions [3, 4]. The ligand v (N=N) value is negatively shifted (20-30 cm^-1^), indicative of azo group coordination. In the FTIR spectrum of copper complex, the absence of the O-H vibrations and the replacement of the C=O vibration by a new band suggests the involvement of the carboxylate group in complex formation. The observed FTIR shifts in the secondary amine vibrations in the zincon reagent to lower values of the v (N-H) and *v*(C=N) band is consistent with metal attachment via nitrogen of the amino group [2, 4] resultant formation of 6-membered ring chelate ring. The v(C=N) band was found in the same position in complexes indicating no participation in the complex formation with Cu^2+^ions.

The reflectance electronic spectrum of Cu^2+^complex shows a broad band cantered at 17241cm^- 1^ interpretable in terms of square planar stereochemistry [5]. Based on these data and the electronic spectra, it can be concluded that, the zincon reagent coordinated to Cu^2+^ via two N and two O of the azo and phenolic OH groups. Thus, the chemical structure of the produced copper-zincon complex can be postulated as [CuL_2_] where L= zincon. Thus, the most probable structure of Cu^2+^–zincon chelate can be demonstrated as in ESI. 1.


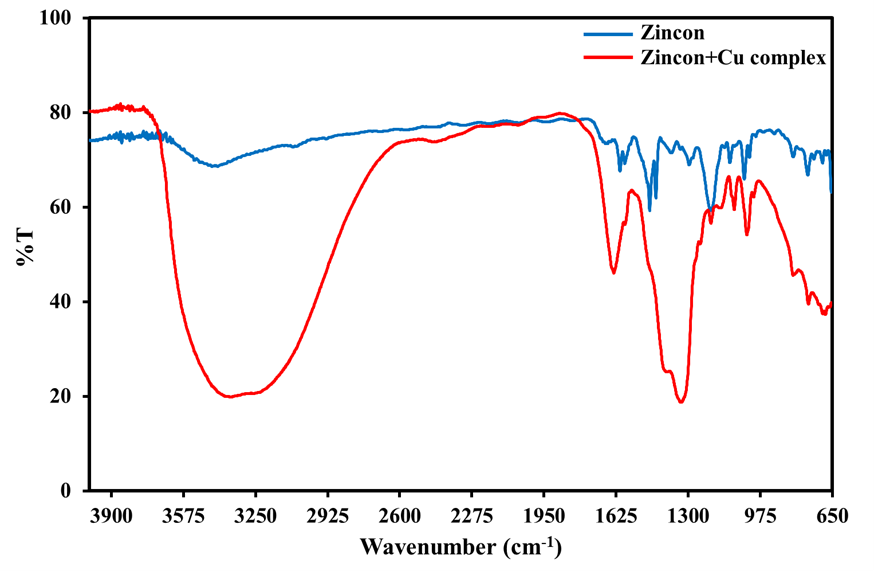


**ESI.6.** FTIR spectra of zincon and its Cu^2+^ complex.

**References**

[1] D. T. Sawyer, W.R. Heinemann, J.M. Beebe, Chemistry Experiments for Instrumental Methods, John Wiley & Sons, 1984.

[2] A.Kocyła, Adam Pomorski, ArturKrężel,Molar absorption coefficients and stability constants of Zincon metal complexes for determination of metal ions and bioinorganic applications, Journal of Inorganic Chemistry, 176 (2017) 53- 65.

[3] Z. Marczenko, Separation and Spectrophotometric Determination of Elements, 2^nd^edn., John Wiley & Sons, 1986.

[4] K. Nakamoto, Infrared and Raman Spectra of Inorganic and Coordination Compounds, Wiley Interscience, New York, 1971.

[5] A.B.P. Lever, Inorganic Electronic Spectroscopy, Theory and Applications, 2 edition, Elsevier Science, Amsterdam, 1984.
